# A cohort study on the biochemical and haematological parameters of Italian blood donors as possible risk factors of COVID-19 infection and severe disease in the pre- and post-Omicron period

**DOI:** 10.1371/journal.pone.0294272

**Published:** 2023-11-21

**Authors:** Chiara Marraccini, Lucia Merolle, Davide Schiroli, Agnese Razzoli, Gaia Gavioli, Barbara Iotti, Roberto Baricchi, Marta Ottone, Pamela Mancuso, Paolo Giorgi Rossi

**Affiliations:** 1 Transfusion Medicine Unit, Azienda USL-IRCCS di Reggio Emilia, Reggio Emilia, Italy; 2 Clinical and Experimental Medicine PhD Program, University of Modena and Reggio Emilia, Reggio Emilia, Italy; 3 Epidemiology Unit, Azienda USL-IRCCS di Reggio Emilia, Reggio Emilia, Italy; University of Bologna / Romagna Local Health Authority, ITALY

## Abstract

To investigate the association between biochemical and blood parameters collected before the pandemic in a large cohort of Italian blood donors with the risk of infection and severe disease. We also focused on the differences between the pre- and post-Omicron spread in Italy (i.e., pre- and post-January 01, 2022) on the observed associations. We conducted an observational cohort study on 13750 blood donors was conducted using data archived up to 5 years before the pandemic. A t-test or chi-squared test was used to compare differences between groups. Hazard ratios with 95% confidence intervals for SARS-CoV-2 infection and severe disease were estimated using Cox proportional hazards models. Subgroup analyses stratified by sex, age and epidemic phase of first infection (pre- and post-Omicron spread) were examined. We confirmed a protective effect of groups B and O, while groups A and AB had a higher likelihood of infection and severe disease. However, these associations were only significant in the pre-Omicron period. We found an opposite behavior after Omicron spread, with the O phenotype having a higher probability of infection. When stratified by variant, A antigen appeared to protect against Omicron infection, whereas it was associated with an increased risk of infection by earlier variants. We were able to stratify for the SARS CoV-2 dominant variant, which revealed a causal association between blood group and probability of infection, as evidenced by the strong effect modification observed between the pre- and post-Omicron spread. The mechanism by which group A acts on the probability of infection should consider this strong effect modification.

## Introduction

Since the COVID-19 pandemic outbreak in early 2020, many studies have attempted to correlate the risk of developing severe symptoms with a heterogeneous range of parameters. Age, pre-existing conditions, red blood cells (RBC), and platelet count (PLT) have been immediately proposed as factors significantly influencing COVID-19 severity [1]. Unexpectedly, other less evident features have emerged as possible prognostic factors: among these, ABO blood group has been associated with both the risk of infection and disease severity [2, 3], while the RBCs distribution width (RDW) has been strongly correlated with disease severity and mortality [3, 4].

Although essential for assessing potential transmission in new areas and preventing viral spread [5], identifying factors that determine the risk of SARS-CoV-2 infection is more complicated than identifying prognostic factors. The reason lies mainly in the lack of a large representative sample of the general population for which we have accessible clinical data in the period immediately preceding (1–2 years) SARS-CoV-2 spread. Hospital-based clinical databases can be used to construct retrospective cohort studies, but they generally refer to individuals suffering from various diseases that are not representative of the general population. In this sense, the UK Biobank (https://www.ukbiobank.ac.uk) [6], which collects the baseline and clinical records of biological and medical data from half a million people aged 40–69 recruited between 2006 and 2010, is a unique and valuable source of information and has been extensively used to screen for possible markers of COVID-19 severity and risk of infection [7]. Albeit important, the UK Biobank is limited to middle-aged and elderly individuals and mainly refers to values collected at the baseline (e.g., more than ten years ago).

An alternative source of information may be the blood donor registries being developed worldwide by local Transfusion Medicine Units. Despite the arrival of the pandemic deeply impacted donor recruitment routine activity [8], blood donor registries have been immediately exploited to identify possible correlations of the ABO blood group with COVID-19 severity and risk of infection [9–11]. Despite the mechanisms underlying the relationship between ABO blood group and susceptibility to infection are not fully understood, there is accumulating evidence that blood group A individuals are at higher risk of infection than non-A individuals, whereas O group individuals are least susceptible to infection [9]. However, the level of evidence is low, and these observations need further validation [9, 12]. Few studies have attempted to correlate Rh [10] and other antigens [11] with COVID-19 progression and severity. Other parameters routinely collected from blood donors (such as blood count, haemoglobin, cholesterol metabolism, iron metabolism, creatinine, aminotransferases, glycaemia and body mass index) have been poorly studied as well.

We aimed to investigate the association between biochemical and blood parameters collected before the pandemic in a large cohort of blood donors with the risk of infection and severe disease. We also focused on the differences between the pre- and post-Omicron period on the observed associations.

## Materials and methods

### Study population and study setting

This is a retrospective cohort study on blood donors of the Reggio Emilia province, in Northern Italy. Reggio Emilia has 531,751 inhabitants and there are six hospitals. By February 28, 2022 there were 121,154 ascertained cases of COVID-19 (22.5% of the total Reggio Emilia population) and a 60% three-doses vaccination coverage [13].

Six donor centres are located within the hospitals, and 21 collection points are run by the local voluntary association. Reggio Emilia accounts for over 14500 periodic blood donors, which must satisfy specific requirements: age between 18 and 65 years; weight ≥ 50 kg; systolic blood pressure ≤ 180 mmHg; diastolic blood pressure ≤ 100 mmHg; regular cardiac pulses (between 50 and 100 beats/min); Hb ≥ 13.5 g/dl for men and ≥ 12.5 g/dl for women [14]. Periodic donors were defined as those that donated at least once every two years in the five years pre-pandemic considered, and perform at least once a year: blood pressure, glycaemia, creatinine, alanine-amino-transferase (ALT), total cholesterol, HDL, LDL, triglycerides, total proteins and ferritin; and at each donation: complete blood count, HbsAg, Anti-HCV antibodies, HIV test, Anti-Treponema Palladium antibodies, HCV, HBV and HIV NAT, ABO, Rh and Kell antigens. Weight, height and body mass index (BMI) are also collected periodically. ABO, Rh and Kell are re-assessed at every donation, while complete phenotype characterisation (which includes Duffy, Kidd, MNS and Cw antigens) is performed only on O-group donors at their first donation. According to our Transfusion Medicine Unit protocols, blood grouping was performed on fully automated immunohematology system Galileo Neo (Immucor Inc., Norcross, GA, USA) using commercially available antisera’s (Immucor Inc., Norcross, GA, USA). Phenotyping was based on Capture technology (solid phase adherence).

Only periodic donors already active before the pandemic and their blood test data performed between February 1, 2015 and January 31, 2020, were included in the study.

In this study, the pre-Omicron period corresponds to the timeframe between February 20, 2020 and December 20, 2021, when the Emilia-Romagna region faced three main COVID-19 waves, driven by the SARS-CoV-2 wild-type, the Alpha (Pango lineage designation B.1.1.7), and Delta (Pango lineage designation B.1.617.2) variant [12]; the Omicron period corresponds to the timeframe between from January 01, 2022 and February 28, 2022, represented by the fourth wave driven by the Omicron BA.1 variant [12].

### Data source

Data concerning the Transfusion Medicine Unit procedures and blood tests, performed on periodic donors, were collected from the internal database (Eliot, Engineering Ingegneria Informatica S.p.A., Italy), which is the central donor registry to which every blood centre of the Reggio Emilia province collects the information concerning donors and donations. The database was accessed on 14 July 2022. Date of symptom onset, diagnosis, hospitalization, and death were retrieved from the COVID-19 Surveillance Registry, implemented in each Local Health Authority. Data from the COVID-19 Surveillance Registry were accessed on 25 July 2022 and linked with the blood test data. Only the PI and co-PI accessed the databases, and all data on recruited donors were anonymised before the analysis. Outcomes of interest were: being tested for SARS-CoV-2 (at least one RT-PCR or antigen test performed), resulting positive for SARS-CoV-2 (at least one test resulted positive), and severe disease (SARS-CoV-2 infection followed by COVID-19 leading to hospitalization within 28 days from diagnosis or death within 90 days). The study follow-up started from February 20, 2020, and ended on February 28, 2022.

### Statistical analyses

Continuous variables are reported as mean and standard deviation (SD) and categorical variables as proportions. With regard to the variables collected several times for the same donor during the 5 years preceding the pandemic, the mean value was taken into account. The inter-group differences were compared by using a t-test or chi-square test. Cox proportional hazard models were used to estimate hazard ratios (HR) with 95% confidence intervals (95% CI) for SARS-CoV-2 infection and for severe disease. For continuous variables, the HRs are computed for an increase from 25 to 75 centile of the exposure variable. For variables showing association, we also explored some subgroup analysis stratifying by sex, age (<50 and > = 50), and epidemic phase of first infection (pre-Omicron period, i.e., from February 20, 2020 to December 20, 2021 and Omicron period, i.e. from January 01, 2022 to February 28, 2022). Given the descriptive nature and hypothesis generation scope of the study, we did not fix a significance threshold. Confidence intervals should be interpreted as a description of the precision of the estimate, as well as p-values should be considered as continuous variables, not as formal statistical tests. Models were adjusted for age, sex, and vaccination history. STATA v. 16.0 was used for all analyses.

### Ethics approval

The Ethics Committee of the Area Vasta Emilia Nord approved the study on 8 September 2021 (no. 2021/0111394). The Ethics Committee exempted the investigators from the obligation to collect written informed consent from all donors, the consent was asked only to those who attended a blood donation during the recruitment period (from 2 May 2022 to 1 June 2022). All donors in the province of Reggio Emilia were also informed of the study with a personal e-mail and SMS, through the usual communication channels used to advise donors by the local AVIS association personnel, and the opportunity to opt-off was offered simply by answering with an SMS or e-mail. The data from donors who chose not to participate either by refusing to sign the consent or by requesting an opt-off were not used.

## Results

Starting from the 14,445 donors attending Reggio Emilia donor centres (and after the exclusion of non-periodic donors), 13750 participants were included in the study (Table 1). Of these, 9528 were males and 4222 were females. By February 28, 2022 (which corresponds to the end of the post-Omicron period considered in this study), 864 donors were still unvaccinated (6.3% of the total), while 9591 (69.7%) had already received three doses. A total number of 7976 donors (58%) were tested for SARS-CoV-2 infection and positivity was registered for 3690 donors (26.8%): of these, 73 (0.5%) experienced the severe disease, which led to hospitalisation and/or death. Infections were equally distributed between pre- and post-Omicron spread (1189 vs. 1169, S2 Table), while all the severe diseases occurred in the pre-Omicron period.

**Table 1:** Demographic data of the cohort of periodic donors, and their association with SARS-CoV-2 testing, SARS-CoV-2 positivity, and COVID-19 severe disease.

	Donors	% / mean (sd)	Tested for SARS-CoV-2					Positivity to SARS-CoV-2					Severe disease				
			Yes	% / mean (sd)	No	% / mean (sd)	p-value	Positive	% / mean (sd)	Negative	% /mean (sd)	p-value	Yes	% / mean (sd)	No	% / mean (sd)	p-value
**Overall**	**13750**		**7976**	**58.0**	**5774**	**42.0**		**3690**	**26.8**	**10060**	**73.2**		**73**	**0.5**	**13677**	**99.5**	
Males	9528	69.3	5395	67.6	4133	71.6	0.000	2574	69.8	6954	69.1	0.477	56	76.7	9472	69.3	0.168
Females	4222	30.7	2581	32.4	1641	28.4		1116	30.2	3106	30.9		17	23.3	4205	30.7	
Age	13750	44.1 (13.1)	7976	42.7 (12.8)	5774	46.1 (13.0)	0.000	3690	42.2 (12.3)	10060	44.8 (13.2)	0.000	73	54.6 (7.5)	13677	44.1 (13.0)	0.000
Weight (kg)	13418	77.6 (14.0)	7798	77.5 (14.3)	5620	77.7 (13.7)	0.513	3606	78.1 (14.2)	9812	77.4 (14.0)	0.019	70	86.2 (13.5)	13348	77.5 (14.0)	0.000
Dyastolic pressure (mmHg)	13615	79.3 (7.9)	7912	78.9 (7.8)	5703	79.8 (7.9)	0.000	3662	78.9 (7.9)	9953	79.4 (7.9)	0.001	72	82.6 (7.1)	13543	79.3 (7.9)	0.000
Systolic pressure (mmHg)	13617	123.4 (12.3)	7912	122.6 (11.8)	5705	124.6 (12.8)	0.000	3662	122.5 (11.7)	9955	123.8 (12.4)	0.000	72	128.1 (12.0)	13545	123.4 (12.2)	0.001
**Vaccination status**																	
Unvaccinated	864	6.3	537	6.7	327	4.1	0.000	379	10.3	485	4.8	0.000	14	19.2	850	6.2	0.000
Vaccinated with one dose	217	1.6	199	2.5	18	0.2		182	4.9	35	0.3		5	6.8	212	1.6	
Vaccinated with two doses	3,078	22.4	2460	30.8	618	7.7		1969	53.4	1109	11.0		30	41.1	3048	22.3	
Vaccinated with three doses	9,591	69.8	4780	59.9	4811	60.3		1160	31.4	8431	83.8		24	32.9	9567	69.9	
**Blood count**																	
RBC (10^6^/μl)	13750	4.9 (0.4)	7976	4.9 (0.4)	5774	4.9 (0.4)	0.137	3690	4.9 (0.4)	10060	4.9 (0.4)	0.602	73	5.0 (0.4)	13677	4.9 (0.4)	0.102
HGB (g/dL)	13750	14.7 (1.2)	7976	14.7 (1.2)	5774	14.8 (1.2)	0.000	3690	14.7 (1.2)	10060	14.8 (1.2)	0.435	73	15.0 (1.1)	13677	14.8 (1.2)	0.097
HCT (%)	13750	43.4 (3.3)	7976	43.3 (3.3)	5774	43.6 (3.2)	0.000	3690	43.3 (3.3)	10060	43.5 (3.3)	0.080	73	44.2 (2.9)	13677	43.4 (3.3)	0.038
MCV (fL)	13750	88.3 (4.1)	7976	88.1 (4.1)	5774	88.5 (4.1)	0.000	3690	88.0 (3.9)	10060	88.4 (4.1)	0.000	73	88.4 (4.0)	13677	88.3 (4.1)	0.828
MCH (pg)	13750	30.0 (1.7)	7976	29.9 (1.7)	5774	30.1 (1.7)	0.000	3690	29.9 (1.6)	10060	30.0 (1.7)	0.051	73	29.9 (1.8)	13677	30.0 (1.7)	0.812
MCHC (g/dL)	13750	34.0 (1.1)	7976	34.0 (1.1)	5774	34.0 (1.1)	0.339	3690	34.0 (1.0)	10060	34.0 (1.1)	0.022	73	33.9 (1.2)	13677	34.0 (1.1)	0.446
RDW (%)	13750	11.7 (0.8)	7976	11.7 (0.8)	5774	11.7 (0.8)	0.031	3690	11.7 (0.8)	10060	11.7 (0.8)	0.000	73	11.8 (0.8)	13677	11.7 (0.8)	0.204
PLT (10^3^/μl)	13750	223.7 (46.4)	7976	224.0 (46.6)	5774	223.4 (46.2)	0.509	3690	223.0 (46.1)	10060	224.0 (46.5)	0.246	73	215.2 (44.8)	13677	223.8 (46.4)	0.115
MPV (fL)	13750	7.3 (1.3)	7976	7.3 (1.3)	5774	7.2 (1.2)	0.006	3690	7.3 (1.3)	10060	7.3 (1.2)	0.252	73	7.5 (1.3)	13677	7.3 (1.2)	0.156
**Leucocyte formula**																	
WBC (10^3^/μl)	13750	5.9 (1.4)	7976	5.9 (1.4)	5774	5.9 (1.4)	0.584	3690	5.9 (1.4)	10060	5.9 (1.5)	0.014	73	5.9 (1.4)	13677	5.9 (1.4)	0.709
Neutrophils (10^3^/ml)	13750	3.3 (1.1)	7976	3.3 (1.1)	5774	3.3 (1.1)	0.798	3690	3.2 (1.1)	10060	3.3 (1.1)	0.002	73	3.2 (1.0)	13677	3.3 (1.1)	0.630
Lymphocytes (10^3^/ml)	13750	1.9 (0.5)	7976	1.9 (0.5)	5774	1.9 (0.5)	0.091	3690	1.9 (0.5)	10060	1.9 (0.5)	0.652	73	1.9 (0.5)	13677	1.9 (0.5)	0.966
Monocytes (10^3^/μl)	13750	0.5 (0.1)	7976	0.5 (0.1)	5774	0.5 (0.1)	0.274	3690	0.5 (0.1)	10060	0.5 (0.1)	0.212	73	0.5 (0.2)	13677	0.5 (0.1)	0.854
Eosinophils (10^3^/μl)	13750	0.2 (0.1)	7976	0.2 (0.1)	5774	0.2 (0.1)	0.304	3690	0.2 (0.1)	10060	0.2 (0.1)	0.534	73	0.2 (0.1)	13677	0.2 (0.1)	0.970
Basophils (10^3^/ml)	13750	0.07 (0.04)	7976	0.07 (0.04)	5774	0.07 (0.04)	0.013	3690	0.07 (0.03)	10060	0.07 (0.04)	0.093	73	0.07 (0.04)	13677	0.07 (0.04)	0.517
**Blood parameters**																	
Total Protein (g/dL)	13747	7.1 (0.4)	7974	7.1 (0.4)	5773	7.1 (0.4)	0.004	3689	7.2 (0.4)	10058	7.1 (0.4)	0.000	73	7.1 (0.4)	13674	7.1 (0.4)	0.185
Total Colesterol (mg/dL)	13746	195.7 (34.6)	7974	194.2 (34.7)	5772	198.0 (34.2)	0.000	3689	193.9 (34.9)	10057	196.4 (34.4)	0.000	73	209.6 (33.4)	13673	195.7 (34.5)	0.001
Colesterol HDL (mg/dL)	13243	57.7 (13.4)	7644	57.4 (13.3)	5599	58.0 (13.5)	0.030	3546	57.0 (13.2)	9697	57.9 (13.4)	0.001	71	53.8 (13.2)	13172	57.7 (13.4)	0.016
Triglycerides (mg/dL)	13746	95.3 (59.9)	7974	95.2 (62.3)	5772	95.6 (54.6)	0.681	3689	95.7 (63.5)	10057	95.2 (58.5)	0.672	73	115.3 (54.9)	13673	95.2 (59.9)	0.004
Ferritin (ng/ml)	13746	56.1 (48.1)	7974	55.9 (48.8)	5772	56.3 (47.3)	0.578	3689	57.2 (51.3)	10057	55.7 (46.9)	0.103	73	61.5 (44.8)	13673	56.0 (48.2)	0.329
Glycaemia (mg/dL)	13745	89.1 (12.1)	7973	88.5 (11.3)	5772	89.8 (13.1)	0.000	3689	88.4 (10.6)	10056	89.4 (12.6)	0.000	73	95.0 (16.1)	13672	89.1 (12.1)	0.000
ALT/SGPT (U/L)	13747	25.0 (11.1)	7974	25.0 (12.0)	5773	24.9 (13.5)	0.725	3689	25.1 (11.1)	10058	24.9 (13.2)	0.444	73	27.4 (11.1)	13674	25.0 (12.7)	0.105
Creatinin (mg/dL)	13746	0.9 (0.1)	7974	0.9 (0.1)	5772	0.9 (0.1)	0.005	3689	0.9 (0.1)	10057	0.9 (0.1)	0.025	73	0.9 (0.2)	13673	0.9 (0.1)	0.076
**ABO**																	
0	6280	45.7	3652	45.8	2628	45.5	0.981	1684	45.6	4596	45.7	0.914	23	31.5	6257	45.7	0.051
A	5466	39.8	3156	39.6	2310	40.0		1480	40.1	3986	39.6		41	56.2	5425	39.7	
AB	576	4.2	337	4.2	239	4.1		147	4.0	429	4.3		4	5.5	572	4.2	
B	1402	10.2	815	10.2	587	10.2		371	10.1	1031	10.2		5	6.8	1397	10.2	
Missing	26	0.2	16	0.2	10	0.2		8	0.2	18	0.2		0	0.0	26	0.2	
**Rh**																	
Neg	2062	15.0	1198	15.0	864	15.0	0.868	560	15.2	1502	14.9	0.887	9	12.3	2053	15.0	0.755
Pos	11661	84.8	6761	84.8	4900	84.9		3122	84.6	8539	84.9		64	87.7	11597	84.8	
Missing	27	0.2	17	0.2	10	0.2		8	0.2	19	0.2		0	0.0	27	0.2	
**Rh blood group antigens**																	
CCDEe	30	0.2	20	0.3	10	0.2	0.670	13	0.4	17	0.2	0.457	0	0.0	30	0.2	0.968
CCDee	3244	23.6	1926	24.1	1318	22.8		879	23.8	2365	23.5		16	21.9	3228	23.6	
CCDuee	2	0.0	1	0.0	1	0.0		0	0.0	2	0.0		0	0.0	2	0.0	
CcDEE	9	0.1	4	0.1	5	0.1		0	0.0	9	0.1		0	0.0	9	0.1	
CcDEe	1644	12.0	958	12.0	686	11.9		437	11.8	1207	12.0		10	13.7	1634	11.9	
CcDee	4874	35.4	2790	35.0	2084	36.1		1280	34.7	3594	35.7		26	35.6	4848	35.4	
CcDuee	60	0.4	31	0.4	29	0.5		13	0.4	47	0.5		0	0.0	60	0.4	
CcdEe	2	0.0	2	0.0	0	0.0		1	0.0	1	0.0		0	0.0	2	0.0	
Ccdee	86	0.6	55	0.7	31	0.5		25	0.7	61	0.6		0	0.0	86	0.6	
ccDEE	194	1.4	110	1.4	84	1.5		63	1.7	131	1.3		1	1.4	193	1.4	
ccDEe	1291	9.4	733	9.2	558	9.7		350	9.5	941	9.4		10	13.7	1281	9.4	
ccDee	303	2.2	181	2.3	122	2.1		85	2.3	218	2.2		1	1.4	302	2.2	
ccDuEe	3	0.0	2	0.0	1	0.0		0	0.0	3	0.0		0	0.0	3	0.0	
ccDuee	6	0.0	4	0.1	2	0.0		1	0.0	5	0.0		0	0.0	6	0.0	
ccdEe	35	0.3	24	0.3	11	0.2		10	0.3	25	0.2		1	1.4	34	0.2	
Ccdee	1938	14.1	1116	14.0	822	14.2		524	14.2	1414	14.1		8	11.0	1930	14.1	
Missing	29	0.2	19	0.2	10	0.2		9	0.2	20	0.2		0	0.0	29	0.2	
**Kell**																	
K+	29	0.2	14	0.2	15	0.3	0.248	6	0.2	23	0.2	0.618	0	0.0	29	0.2	0.224
KK	27	0.2	19	0.2	8	0.1		10	0.3	17	0.2		0	0.0	27	0.2	
Kk	1115	8.1	640	8.0	475	8.2		288	7.8	827	8.2		5	7.0	1110	8.1	
kk	12493	90.9	7246	90.8	5247	90.9		3362	91.1	9131	90.8		66	93.0	12427	90.9	
Missing	86	0.6	57	0.7	29	0.5		24	0.7	62	0.6		0	0.0	86	0.6	
**Cw** *																	
Cw+	104	0.8	61	0.8	43	0.7	0.923	28	0.8	76	0.8	0.742	0	0.0	104	0.8	0.616
Cw-	4785	34.8	2765	34.7	2020	35.0		1265	34.3	3520	35.0		23	31.5	4762	34.8	
**MN** *																	
MN	3387	24.6	1932	24.2	1455	25.2	0.008	899	24.4	2488	24.7	0.094	16	21.9	3371	24.6	0.598
NN	1384	10.1	763	9.6	621	10.8		351	9.5	1033	10.3		5	6.8	1379	10.1	
MM	2268	16.5	1297	16.3	971	16.8		579	15.7	1689	16.8		11	15.1	2257	16.5	
**Ss**																	
SS	973	7.1	564	7.1	409	7.1	0.003	266	7.2	707	7.0	0.003	5	6.8	968	7.1	0.525
Ss	3222	23.4	1784	22.4	1438	24.9		782	21.2	2440	24.3		15	20.5	3207	23.4	
Ss	2778	20.2	1610	20.2	1168	20.2		763	20.7	2015	20.0		11	15.1	2767	20.2	
**Duffy** *																	
Fya-b+	2578	18.7	1463	18.3	1115	19.3	0.027	662	17.9	1916	19.0	0.209	17	23.3	2561	18.7	0.320
Fya+b+	3088	22.5	1758	22.0	1330	23.0		803	21.8	2285	22.7		10	13.7	3078	22.5	
Fya+b-	1293	9.4	727	9.1	566	9.8		344	9.3	949	9.4		5	6.8	1288	9.4	
Fya-b-	27	0.2	19	0.2	8	0.1		6	0.2	21	0.2		0	0.0	27	0.2	
**Kidd** *																	
Jka-b+	1700	12.4	962	12.1	738	12.8	0.035	426	11.5	1274	12.7	0.094	12	16.4	1688	12.3	0.374
Jka+b+	3492	25.4	1973	24.7	1519	26.3		916	24.8	2576	25.6		12	16.4	3480	25.4	
Jka+b-	1781	13.0	1018	12.8	763	13.2		464	12.6	1317	13.1		8	11.0	1773	13.0	
Jka-b-	2	0.0	1	0.0	1	0.0		0	0.0	2	0.0		0	0.0	2	0.0	

NOTES

* Tested only in O-group donors

*Tested for SARS-CoV-2*: donors having at least one RT-PCR or antigen test for SARS-CoV-2 performed

*Positivity to SARS-CoV-2*: donors having at least one test resulted positive for SARS-CoV-2

*Severe disease*: donors with COVID-19 disease leading to hospitalization within 28 days from diagnosis or death within 90 days

*% / mean (sd)*: the values in the column are expressed as % (sd) for the categorical variables and mean (sd) for the continuous variables.

### SARS-CoV-2 risk of infection

**Fig 1:**
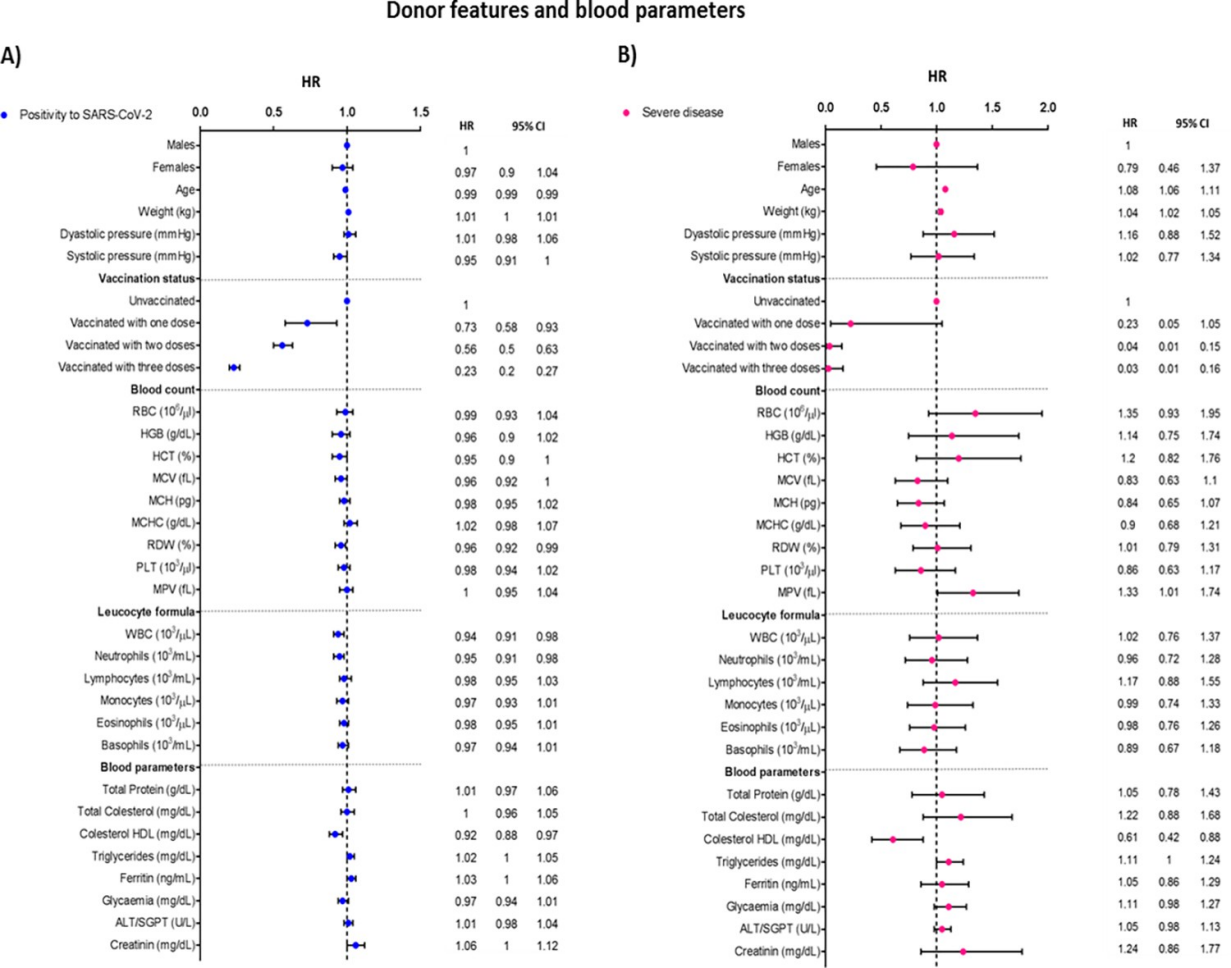
Cox proportional regression analysis adjusted by age, sex, and vaccination status, according to donor features and blood parameters.

In the overall donor population, vaccination was associated to a lower probability of infection at increasing doses, with an almost null risk of severe disease at three doses (Fig 1 and S1 Table).

**Fig 2:**
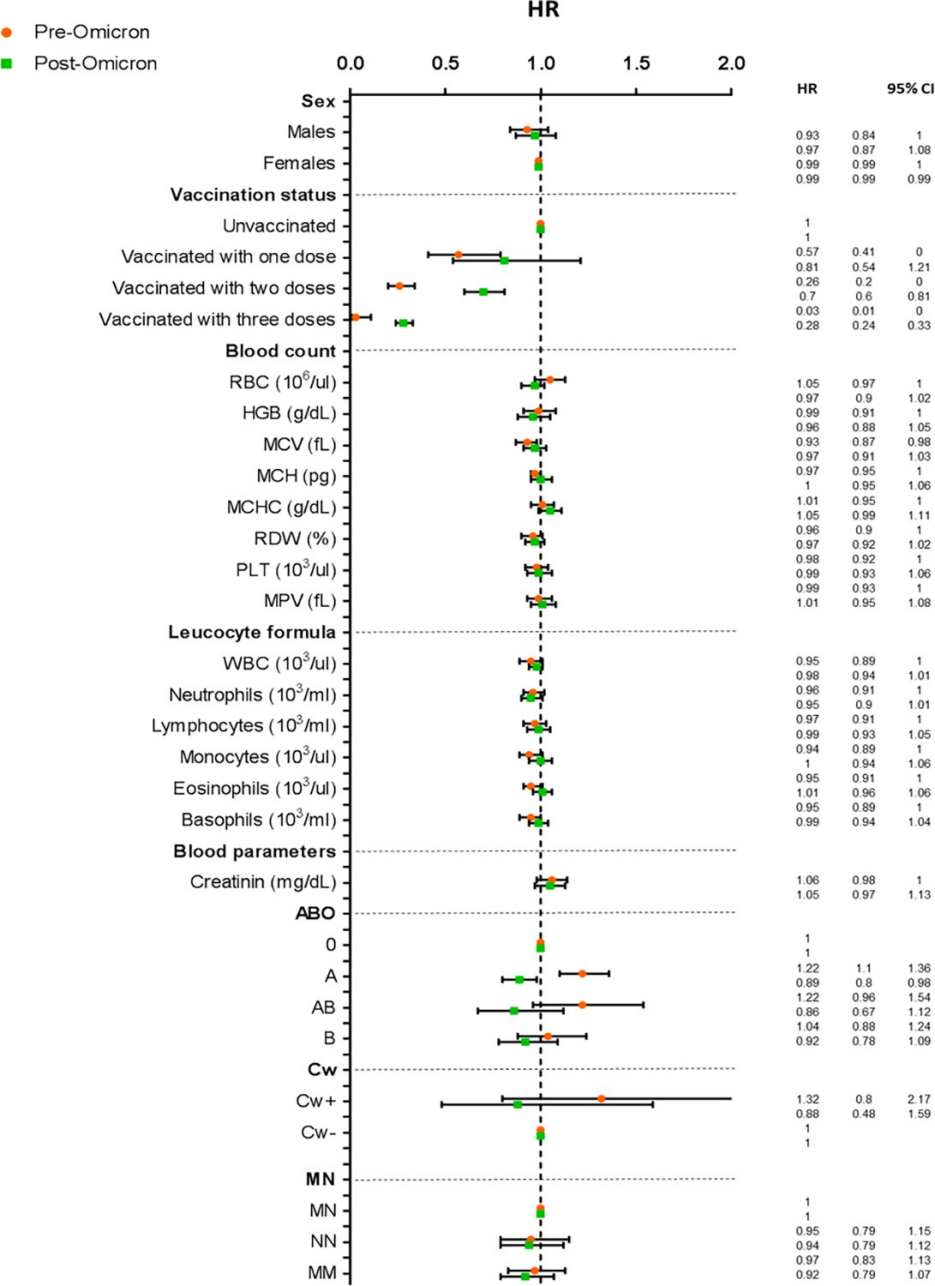
Cox proportional regression analysis adjusted by sex, age and vaccination status in donors tested positive for SARS-CoV-2 before (pre-Omicron diffusion) and after December 20, 2021 (post-Omicron diffusion).

The protection of vaccines against the risk of infection decreased after Omicron diffusion (Fig 2 and S2 Table), for which on average the same protection was reached with at least one more dose than before. The protection against the risk of infection given by the vaccination was more evident in donors above the age of 50 (S4 Table).

High RDW was found to be weakly associated with decreased risk of infection (HR = 0.96, 95% CI 0.92 to 0.99, Fig 1 and S1 Table). This association appears to be mainly determined by the over-50 donor population (HR = 0.93, 95% CI 0.87 to 0.99, S4 Table).

The increase in the total amount of white blood cells (WBCs) was weakly associated with the risk of infection (HR = 0.95, 95% CI 0.89 to 1.01, Fig 1 and S1 Table); nevertheless, the difference was compatible with chance. The association was stronger in donors over 50 years of age (HR = 0.88, 95% CI 0.81 to 0.95, S4 Table). Among the leucocyte subpopulations monocytes, high levels of eosinophils and basophils were inversely associated with infection with the pre-Omicron period (HR = 0.94 for monocytes, HR = 0.95 for eosinophils and basophils, Fig 2 and S2 Table), while after Omicron spread only the association is only with neutrophils subpopulation (HR = 0.95, 95% CI 0.90 to 1.01, Fig 2 and S2 Table). It is worth noting that all these associations are compatible with chance.

Among blood parameters, high creatinine was associated with an increased risk of infection overall (HR = 1.06, 95% CI 1.00 to 1.12), in males (S3 Table), and over the age of 50 (S4 Table), whereas, again, we observed no major differences when looking at the Omicron variant (Fig 2). Conversely, high HDL cholesterol was associated with a lower risk of SARS-Cov-2 infection (HR = 0.92, 95% CI 0.88 to 0.97, S1 Table and Fig 1).

**Fig 3:**
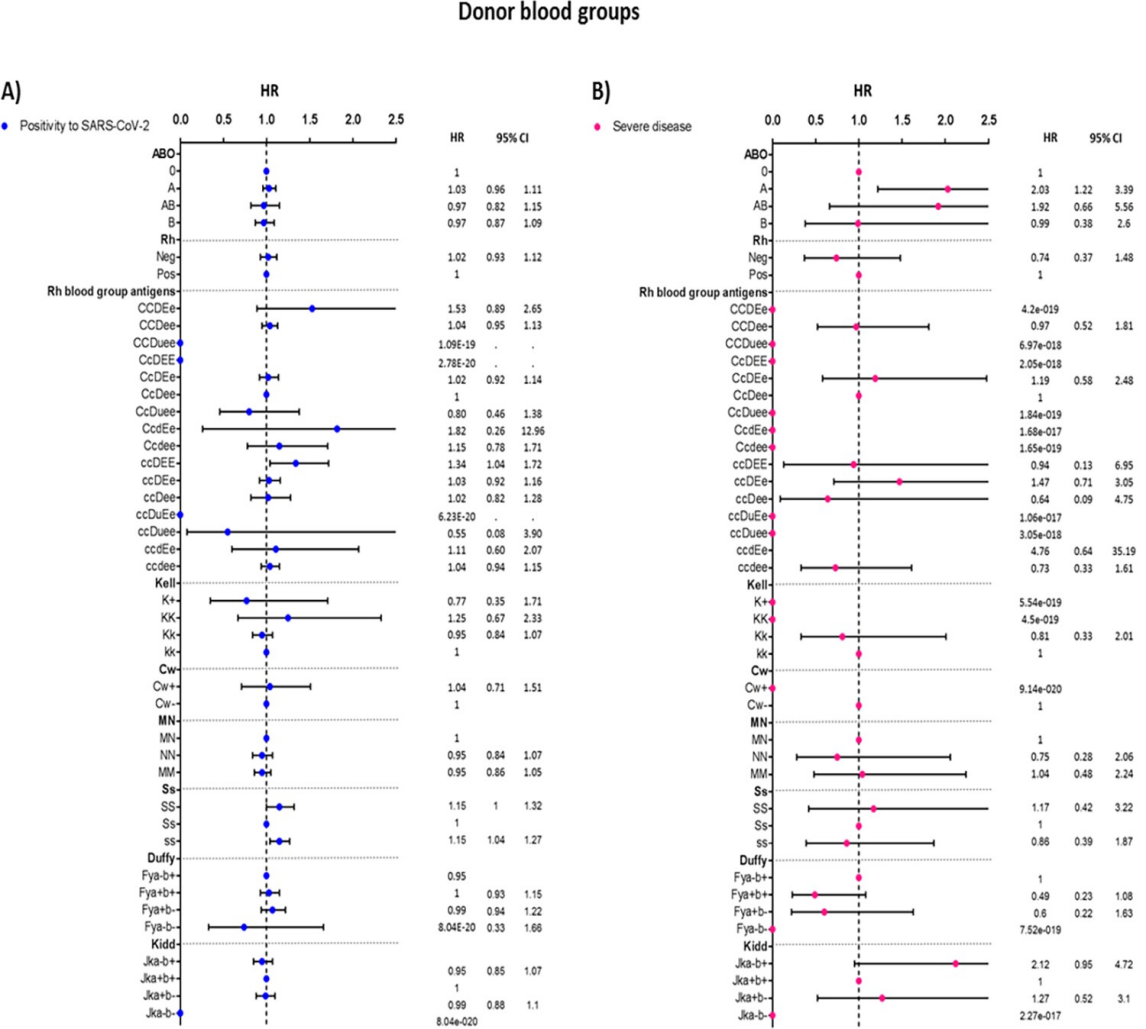
Cox proportional regression analysis adjusted by age, sex, and vaccination status, according to donor blood groups.

The ABO blood group was not associated with SARS-CoV-2 positivity in the whole study period (Fig 3). The association with infection emerged after stratifying by period: in the pre-Omicron period, the A and AB groups were associated with an increased risk of infection, while post-Omicron spread the O group showed increased risk compared to B, AB, and mostly A. Among the Rh antigens, the ccDEE was associated with higher incidence (HR 1.34, 95% CI 1.04 to 1.72). Among the genes, Ss heterozygotes showed a lower probability of infection.

### Risk of COVID-19 severe disease

The severe disease mainly occurred in older donors (mean age 54.6±7.5 vs 44.1±13.0 in the whole donor population), on average 8.7 kg heavier (86.2±13.5 vs 77.5±14.0 kg) and with slightly higher blood pressure (both systolic and diastolic, Table 1). The Cox model adjusted for sex, age, and vaccination history (Fig 1 and S1 Table) confirmed an independent association with COVID-19 severity only for age, weight (HR 1.08 per year increase, 95%CI 1.06–1.11; and 1.04 per kg increase, 95%CI 1.02–1.05), and vaccination.

High RBCs (Table 1) were associated with the risk of severe disease but the estimate was extremely imprecise (HR = 1.35, 95% CI 0.93 to 1.95, Fig 1). Other red blood cell indexes showed weak and inconsistent associations with severe disease, all compatible with random fluctuations. Mean platelet volume (MPV) was found to behave in the opposite way (Table 1), being its increase associated with higher risk (HR = 1.19, 95% CI 1.01 to 1.41, Fig 1 and S1 Table).

Donors that experienced severe disease also showed higher total cholesterol (209.6±33.4 vs 195.7±34.5 mg/dL, Table 1) and triglycerides (115.3±54.9 vs 95.2±59.9 mg/dL, Table 1), whereas HDL was slightly lower (53.8±13.2 vs 57.7±13.4 mg/dL, Table 1). According to the Cox regression model, however, the association with creatinine became extremely imprecise (HR = 2.90, 95% CI 0.48 to 17.41), while the role of total cholesterol and HDL were confirmed.

When considering the totality of recruited subjects, the ABO blood group results are associated with COVID-19 severity (p-value = 0.051), with a higher occurrence of group A among the hospitalized donors. The risk of developing severe disease was significantly higher for donors bearing the A antigen (HR = 2.03 for group A; HR = 1.92 for group AB), compared to those that express only the B antigen or to those with the ABO blood type O (Fig 3 and S1 Table).

Regarding the blood subgroups, estimates are too imprecise to give any suggestion about associations. For many subgroups, zero events were observed. Only for Jka-b+ phenotype an increase in the occurrence of severe disease, based on only 12 cases, was observed but the estimate is very imprecise (HR = 2.12, 95% CI 0.95 to 4.72). Since no hospitalization or death occurred in the recruited population during the Omicron period, it was not possible to conduct stratified analyses on the risk of severe disease.

## Discussion

We examined the association between the demographic and biochemical blood parameters, collected from a cohort of donors up to 5 years before the pandemic, with the probability of testing positive for SARS-CoV-2 and of being hospitalized for COVID-19. Although quite representative of the general population, blood donors have often been shown to deviate from the distribution of the general population due to their healthier condition [15, 16]. This aspect is a major strength of our cohort, as it allowed us to minimize the effect of comorbidities and population heterogeneity.

During the study period, more than half of the cohort was tested and 26% tested positive for SARS-CoV-2 at least once, a rate closely resembling the general population of similar age (24%) [12]. We observed small differences in testing propensity, suggesting that the probability of receiving a COVID-19 diagnosis in our cohort is similar to that of the general population. Infections were evenly distributed between pre- and post-Omicron spread. This was expected because, although the two time periods considered are significantly different (22 months for the pre-Omicron vs. 2 months for the post-Omicron period), the number of infections recorded in the province of Reggio Emilia during the fourth pandemic wave (corresponding to the spread of Omicron BA.1) was the same as, if not greater than, the sum of infections recorded in the previous 3 waves (determined by the spread of the wild-type, Alpha and Delta variants) [13]. In terms of severe disease, we observed only 73 hospitalizations and 1 death, only three hospitalizations occurred in the Omicron period.

We observed a weak protective effect of high RDW and WBCs against infection in the pre-Omicron period. The effect of blood group changed between pre- and post-Omicron diffusion: group A donors had a higher risk of infection in the pre-Omicron period, but a lower risk in the Omicron period. Other parameters showed weak and highly uncertain associations or no association.

To our knowledge, this is the first time that an inverse association between RDW and risk of infection, independently of virus variant, has been observed. Since RDW values are strictly related to erythropoiesis efficiency and can be enhanced by physical exercise or anaemic conditions [17], thus we cannot exclude that the observed association might be confounded by different social behaviours that change the probability of being exposed to the virus.

We observed a weak but consistent inverse association between WBC count and the probability of infection. It is important to note that the association cannot be due to pathologically low levels, as WBCs must be within the normal range to be eligible for donation. All WBC subpopulations go in the same direction, even if the observed differences in probability of infection are very small and each of them compatible with a random fluctuation; nevertheless, the whole pattern suggests that it is difficult to identify only one sub-group to explain the association with WBCs. High eosinophils, found in the airways of asthmatic patients, have already been suggested to play a protective role against SARS-CoV-2 infection and COVID-19 severity [18], possibly by counteracting the exacerbated inflammatory response typical of the severe COVID-19 phenotype [19]. Intriguingly, after the diffusion of the Omicron variant, the association with WBC subpopulations is lost, except for neutrophils. This could be due to the different Omicron biology and interaction with the host immune system and respiratory tract [20].

As expected, vaccination was protective against both infection and severe disease. We could not test for a difference before and after the spread of the Omicron variant because no hospitalizations or deaths occurred after 20 December 2021, when most of the cohort had already been vaccinated. Nevertheless, our results confirm the differences in protection against infection observed between the pre- and post-Omicron [21]. We observed higher vaccination protection in older people. This may be because older people (>50) were vaccinated earlier, during the peak of the Alpha variant that reached Italy in spring 2021, while younger people (<50) were vaccinated between summer and autumn, when the peak decreased, and the herd immunity increased. In addition, a larger proportion of the <50 years old were vaccinated during the Omicron period.

With the exception of vaccination, we observed only a weak association with the probability of infection. HDL and systolic pressure were inversely associated, while weight, triglycerides, ferritin, and creatinine high levels were associated positively associated. Subgroup analyses suggest that creatinine is associated only in older males. The estimates are rather precise and we can exclude large effects that were not observed by chance. Consistent with our study, serum creatinine was previously found to be associated with disease severity [22].

Body weight, low HDL cholesterol, and group A, were associated with a higher risk of severe disease. The risk of severe disease was also observed to be directly associated with triglycerides (HR = 1.11, 95% CI 1.00 to 1.24, S1 Table) and inversely associated with HDL cholesterol (HR = 0.61, 95% CI 0.42 to 0.88, S1 Table), as expected in a healthy population cohort.

We confirmed a protective effect of groups B and O [9, 23], while groups A and AB have a higher probability of infection and severe disease only in the pre-Omicron period. Surprisingly, we observed an opposite behaviour of blood groups after Omicron spread, with the O phenotype having a higher probability of infection than A. Since early works on SARS-CoV-2, the ABO group has been extensively investigated for its possible role in COVID-19 susceptibility [24]. Although the majority of studies have demonstrated a protective effect of blood group O compared with non-O on both the risk of infection and disease severity, the results have often been conflicting. These controversial observations may be partly explained by the variable distribution of the ABO phenotypes across geographical areas, human populations [25], and, finally, in light of our results, the dominant viral variants in the study period.

ABO antigens are highly abundant not only on the surface of RBCs but also on the epithelial cells in contact with the external environment [26]. Some authors suggested that A antigens expressed on the viral envelope could be recognized by anti-A antibodies in non-A individuals (and particularly in O phenotype individuals, who have the highest anti-A antibody titre) [27, 28]. Others found a similarity between the glycan structures on the SARS-CoV-2 S protein and the domains recognized by anti-A antibodies and hypothesized that the reduced viral infection may be the result of the antibody-mediated blockade of the interaction between the virus and the angiotensin-converting enzyme 2 (ACE2) receptor, thus preventing entry into the lung epithelium [22, 29]. The spread of the Omicron variant has been favoured by a more efficient transmission capacity compared to the Delta variant [22]. The structural differences between the two variants, together with an altered entry mechanism [19], account for immune and antibody escape and may also explain the different associations of this infection with blood groups.

Similarly, we observed a slight difference in the probability of infection for the ccDEE Rh specific antigen phenotype, even if the p-value reached the 5% threshold usually considered to be statistically significant, we cannot exclude that this was due to chance, given the large number of comparisons made and the fact that this association had previously been excluded in a similar cohort [11]. When examining the MNS blood group, we also observed an association for the S antigen (GYPB gene), for which we observed a borderline statistically significant protective effect of the Ss phenotype compared to both SS and ss, we did not find any other study confirming our observation [11].

The risk of hospitalization observed in this donor cohort (0.5%) is much lower than that observed in the general population of the province of Reggio Emilia during the study period (1.8%) [12]. The low hospitalization rate makes the study underpowered to observe even large differences between groups, and we cannot perform a stratified analysis for the Omicron period, because we have almost no hospitalizations after the decline of the Delta variant.

Our results confirm previous literature observations on women (who have a lower risk of developing severe disease than men) and age, which is the strongest determinant with an 8% increase in the probability of infection per year increase. We also confirmed the association with body weight and total cholesterol, as well as the protective effect of HDL.

While a high post-infection RDW is known to be an independent predictor of worse outcomes in patients with respiratory tract infections and has also been associated with adverse COVID-19 progression [30], our data suggest that RDW values observed prior to disease onset are not associated with a higher risk of severe disease. Therefore, RDW can be considered as a possible prognostic biomarker once the disease symptoms have occurred, but this parameter is not a risk factor.

We also observed an association of the Jka-b+ phenotype with COVID-19 severity. Kidd variants implication in pathogens response has been previously modeled [31], while jka and jkb alleles have been identified as risk and protective, respectively, in bladder cancer [32]. Nevertheless, anti-Jkb antibodies only occur after an alloimmunization, an event that is very rare in a healthy donor population. Furthermore, we do not see any excess risk in Jka+b+ phenotype. Therefore, association cannot be due to a protective effect of anti-Jkb antibodies and chance is a plausible explanation.

## Conclusion

In our study of a blood donor cohort, we had the opportunity to stratify for the SARS-CoV-2 dominant variant, which brought to light a novelty regarding the possible impact of blood type on viral infection. We showed a weak association between the blood group and the probability of infection, which is likely to be causal, both because our results are consistent with previous findings [9] and because we observed a strong effect modification between the pre- and post-Omicron spread. The mechanism by which group A affects the probability of infection should now be considered in light of this strong effect modification.

## Supporting information

S1 Table Cox proportional regression analysis adjusted by age, sex, and vaccination status, according to donor features, blood parameters, and blood group. (DOCX)

S2 Table Cox proportional regression analysis adjusted by sex, age, and vaccination status in donors tested positive for SARS-CoV-2 before and after December 20, 2021 (pre- and post-Omicron diffusion, respectively). (DOCX)

S3 Table Cox proportional regression analysis adjusted by age, sex, and vaccination status in male and female donors. (DOCX)

S4 Table Cox proportional regression analysis adjusted by age, sex, and vaccination status in donors under (<50) and over (> = 50) the age of 50. (DOCX)

## References

[1] Izcovich A, Ragusa MA, Tortosa F, Lavena Marzio MA, Agnoletti C, Bengolea A, et al. Prognostic factors for severity and mortality in patients infected with COVID-19: A systematic review. PLoS One 2020;15:e0241955. doi: 10.1371/journal.pone.0241955 33201896 PMC7671522

[2] Zhao J, Yang Y, Huang H, et al. Relationship between the ABO blood group and the coronavirus disease 2019 (COVID-19) susceptibility. Clinical Infectious Diseases 2021;73:328–31. doi: 10.1093/cid/ciaa1150 32750119 PMC7454371

[3] Muñiz-Diaz E, Llopis J, Parra R, et al. Relationship between the ABO blood group and COVID-19 susceptibility, severity and mortality in two cohorts of patients. Blood Transfusion 2021;19:54. doi: 10.2450/2020.0256-20 33196417 PMC7850930

[4] Lee JJ, Montazerin SM, Jamil A, Jamil U, Marszalek J, Chuang ML, et al. Association between red blood cell distribution width and mortality and severity among patients with COVID‐19: A systematic review and meta‐analysis. J Med Virol 2021;93:2513–22. doi: 10.1002/jmv.26797 33448439 PMC8014709

[5] Kucharski AJ, Russell TW, Diamond C, Liu Y, Edmunds J, Funk S, et al. Early dynamics of transmission and control of COVID-19: a mathematical modelling study. Lancet Infect Dis. 2020 May;20(5):553–558. doi: 10.1016/S1473-3099(20)30144-4 32171059 PMC7158569

[6] Sudlow C, Gallacher J, Allen N, Beral V, Burton P, Danesh J, et al. UK biobank: an open access resource for identifying the causes of a wide range of complex diseases of middle and old age. PLoS medicine 2015;12:e1001779. doi: 10.1371/journal.pmed.1001779 25826379 PMC4380465

[7] Chadeau-Hyam M, Bodinier B, Elliott J, Whitaker MD, Tzoulaki I, Vermeulen R, et al. Risk factors for positive and negative COVID-19 tests: a cautious and in-depth analysis of UK biobank data. International journal of epidemiology 2020;49:1454–67. doi: 10.1093/ije/dyaa134 32814959 PMC7454561

[8] Schiroli D, Merolle L, Molinari G, Di Bartolomeo E, Seligardi D, Canovi L, et al. The impact of COVID-19 outbreak on the Transfusion Medicine Unit of a Northern Italy Hospital and Cancer Centre. Vox Sang 2022;117(2):235–242. doi: 10.1111/vox.13174 34156107 PMC8447465

[9] Franchini M, Cruciani M, Mengoli C, Marano G, Candura F, Lopez N, et al. ABO blood group and COVID-19: an updated systematic literature review and meta-analysis. Blood Transfusion 2021;19:317. doi: 10.2450/2021.0049-21 34059188 PMC8297670

[10] Esref A, Solmaz I, Akkoc H, Donmezdil S, Karahan Z, Safak K, et al. Association between the Rh blood group and the Covid-19 susceptibility. Int J Hematol Oncol 2020;32(2):081–086.

[11] Moslemi C, Saekmose S, Larsen R, Brodersen T, Didriksen M, Hjalgrim H, et al A large cohort study of the effects of Lewis, ABO, 13 other blood groups, and secretor status on COVID-19 susceptibility, severity, and long COVID-19. Transfusion. 2023;63(1):47–58. doi: 10.1111/trf.17170 36271437 PMC9874484

[12] Gutiérrez-Valencia M, Leache L, Librero J, Jerico C, German ME, García-Erce JA. ABO blood group and risk of COVID-19 infection and complications: a systematic review and meta-analysis. Transfusion (Paris) 2022;62:493. doi: 10.1111/trf.16748 34773411 PMC8661771

[13] Vicentini M, Venturelli F, Mancuso P, Bisaccia E, Zerbini A, Massari M, et al. Risk of SARS-CoV-2 reinfection by vaccination status, predominant variant and time from prior infection: a cohort study, Reggio Emilia province, Italy, February 2020 to February 2022. Euro Surveill 2023;28(13):2200494. doi: 10.2807/1560-7917.ES.2023.28.13.2200494 36995374 PMC10064646

[14] Ministero della Salute (Italian Ministry of the Health). Accertamento dei requisiti fisici del donatore ed esami obbligatori ad ogni donazione e controlli periodici. DECRETO 2 novembre 2015: Disposizioni relative ai requisiti di qualita’ e sicurezza del sangue e degli emocomponenti (15A09709) 2015; Allegato IV [in italian].

[15] Golding J, Northstone K, Miller LL, Davey Smith G, Pembrey M. Differences between blood donors and a population sample: implications for case-control studies. Int J Epidemiol. 2013;42(4):1145–56. doi: 10.1093/ije/dyt095 23825379 PMC3781001

[16] Atsma F, Veldhuizen I, Verbeek A, de Kort W, de Vegt F. Healthy donor effect: its magnitude in health research among blood donors. Transfusion. 2011;51(8):1820–8. doi: 10.1111/j.1537-2995.2010.03055.x 21342203

[17] Lippi G, Cervellin G, Sanchis-Gomar F. Red blood cell distribution width: a marker of anisocytosis potentially associated with atrial fibrillation. World J Cardiol 2019;11:292. doi: 10.4330/wjc.v11.i12.292 31908729 PMC6937412

[18] Boechat JL, Wandalsen GF, Kuschnir FC, Delgado L. COVID-19 and pediatric asthma: clinical and management challenges. International journal of environmental research and public health 2021;18:1093. doi: 10.3390/ijerph18031093 33530624 PMC7908623

[19] Carli G, Cecchi L, Stebbing J, Parronchi P, Farsi A. Is asthma protective against COVID‐19? Allergy 2021;76:866. doi: 10.1111/all.14426 32479648 PMC7300712

[20] Carabelli AM, Peacock TP, Thorne LG, Harvey WT, Hughes J, COVID-19 Genomics UK Consortium, et al. SARS-CoV-2 variant biology: immune escape, transmission and fitness. Nat Rev Microbiol 2023;21(3):162–177. doi: 10.1038/s41579-022-00841-7 36653446 PMC9847462

[21] Kirsebom FCM, Andrews N, Stowe J, Toffa S, Sachdeva R, Gallagher E, et al. COVID-19 vaccine effectiveness against the omicron (BA.2) variant in England. Lancet Infect Dis 2022;22(7):931–933. doi: 10.1016/S1473-3099(22)00309-7 35623379 PMC9129256

[22] Alfano G, Ferrari A, Fontana F, Mori G, Ligabue G, Giovanella S, et al. Twenty-four-hour serum creatinine variation is associated with poor outcome in the novel coronavirus disease 2019 (COVID-19) patients. Kidney Res Clin Pract 2021;40(2):231–240. doi: 10.23876/j.krcp.20.177 34162049 PMC8237119

[23] Goel R, Bloch EM, Pirenne F, Al-Riyami AZ, Crowe E, Dau L, et al. ABO blood group and COVID-19: a review on behalf of the ISBT COVID-19 Working Group. Vox Sang 2021;116(8):849–861. doi: 10.1111/vox.13076 33578447 PMC8014128

[24] Matzhold EM, Berghold A, Bemelmans MKB, Banfi C, Stelzl E, Kessler HH, et al. Lewis and ABO histo-blood types and the secretor status of patients hospitalized with COVID-19 implicate a role for ABO antibodies in susceptibility to infection with SARS-CoV-2. Transfusion. 2021;61:2736–2745. doi: 10.1111/trf.16567 34151460 PMC8447157

[25] Pendu JL, Breiman A, Rocher J, Dion M, Ruvoën-Clouet N. ABO Blood Types and COVID-19: Spurious, Anecdotal, or Truly Important Relationships? A Reasoned Review of Available Data. Viruses 2021;13(2):160. doi: 10.3390/v13020160 33499228 PMC7911989

[26] Pereira E, Felipe S, de Freitas R, Araújo V, Soares P, Ribeiro J, et al ABO blood group and link to COVID-19: A comprehensive review of the reported associations and their possible underlying mechanisms. Microb Pathog 2022;169:105658. doi: 10.1016/j.micpath.2022.105658 35764188 PMC9233352

[27] Breiman A, Ruvën-Clouet N, Le Pendu J. Harnessing the natural anti-glycan immune response to limit the transmission of enveloped viruses such as SARS-CoV-2. PLoS Pathog 2020;16(5):e1008556. doi: 10.1371/journal.ppat.1008556 32437478 PMC7241692

[28] Deleers M, Breiman A, Daubie V, Maggetto C, Barreau I, Besse T, et al. Covid-19 and blood groups: ABO antibody levels may also matter. Int J Infect Dis. 2021;104:242–249. doi: 10.1016/j.ijid.2020.12.025 33326874 PMC7832075

[29] Watanabe Y, Allen JD, Wrapp D, McLellan JS, Crispin M. Site-specific glycan analysis of the SARS-CoV-2 spike. Science 2020;369:330–3. doi: 10.1126/science.abb9983 32366695 PMC7199903

[30] Gama S. RDW shows prognostic potential in hospitalized patients with COVID‐19. Journal of Medical Virology 2022;94:3498. doi: 10.1002/jmv.27764 35388503 PMC9088635

[31] Fumagalli M, Cagliani R, Pozzoli U, Riva S, Comi GP, Menozzi G, et al. Widespread balancing selection and pathogen-driven selection at blood group antigen genes. Genome Res 2009;19:199–212. doi: 10.1101/gr.082768.108 18997004 PMC2652214

[32] Aleem A, Akbar Samad AB, Vaqar S. Emerging Variants of SARS-CoV-2 And Novel Therapeutics Against Coronavirus (COVID-19). 2023 Feb 5. In: StatPearls [Internet]. Treasure Island (FL): StatPearls Publishing.

