# Supplementary material for: A cohort study on the biochemical and haematological parameters of Italian blood donors as possible risk factors of COVID-19 infection and severe disease in the pre- and post-Omicron period
Source: PLoS One. 2023 Nov 21;18(11):e0294272. doi: 10.1371/journal.pone.0294272 (PMC10662768; doi:10.1371/journal.pone.0294272)
Supplement: S1 Table — (DOCX) [file pone.0294272.s001.docx]

**S1 Table. Cox proportional regression analysis adjusted by age, sex, and vaccination status, according to donor features, blood parameters and blood group:**

|  | **Period of infection from Feb 20, 2020 to Feb 28, 2022** | | | | | | | | |
| --- | --- | --- | --- | --- | --- | --- | --- | --- | --- |
|  | **Positivity to SARS-CoV-2** | | | |  | **Severe disease** | | | |
|  | **Persons-days** | **HR** | **95% CI** | |  | **Persons-days** | **HR** | **95% CI** | |
| Males | 6524180 | 1 |  |  |  | 7025474 | 1 |  |  |
| Females | 2899205 | 0.97 | 0.90 | 1.04 |  | 3116295 | 0.79 | 0.46 | 1.37 |
| Age | 9423385 | 0.99 | 0.99 | 0.99 |  | 10141769 | 1.08 | 1.06 | 1.11 |
| Weight (kg) | 9423385 | 1.01 | 1.00 | 1.01 |  | 10141769 | 1.04 | 1.02 | 1.05 |
| Dyastolic pressure (8.79 IQR mmHg)** | 9423385 | 1.01 | 0.98 | 1.06 |  | 10141769 | 1.16 | 0.88 | 1.52 |
| Systolic pressure (15 IQR mmHg)** | 9423385 | 0.95 | 0.91 | 1.00 |  | 10141769 | 1.02 | 0.77 | 1.34 |
| **Vaccination status** |  |  |  |  |  |  |  |  |  |
| Unvaccinated | 6341901 | 1 |  |  |  | 6638583 | 1 |  |  |
| Vaccinated with one dose | 532343 | 0.73 | 0.58 | 0.93 |  | 706828 | 0.23 | 0.05 | 1.05 |
| Vaccinated with two doses | 2015004 | 0.56 | 0.50 | 0.63 |  | 2210445 | 0.04 | 0.01 | 0.15 |
| Vaccinated with three doses | 534137 | 0.23 | 0.20 | 0.27 |  | 585913 | 0.03 | 0.01 | 0.16 |
| **Blood count**** |  |  |  |  |  |  |  |  |  |
| RBC (0.6 IQR 10^6^/μl) | 9423385 | 0.99 | 0.93 | 1.04 |  | 10141769 | 1.35 | 0.93 | 1.95 |
| HGB (1.7 IQR g/dL) | 9423385 | 0.96 | 0.90 | 1.02 |  | 10141769 | 1.14 | 0.75 | 1.74 |
| HCT (4.5 IQR %) | 9423385 | 0.95 | 0.90 | 1.00 |  | 10141769 | 1.20 | 0.82 | 1.76 |
| MCV (5 IQR fL) | 9423385 | 0.96 | 0.92 | 1.00 |  | 10141769 | 0.83 | 0.63 | 1.10 |
| MCH (1.9 IQR pg) | 9423385 | 0.98 | 0.95 | 1.02 |  | 10141769 | 0.84 | 0.65 | 1.07 |
| MCHC (1.3 IQR g/dL) | 9423385 | 1.02 | 0.98 | 1.07 |  | 10141769 | 0.90 | 0.68 | 1.21 |
| RDW (0.9 IQR %) | 9423385 | 0.96 | 0.92 | 0.99 |  | 10141769 | 1.01 | 0.79 | 1.31 |
| PLT (59 IQR 10^3^/μL) | 9423385 | 0.98 | 0.94 | 1.02 |  | 10141769 | 0.86 | 0.63 | 1.17 |
| MPV (1.6 IQR fL) | 9423385 | 1.00 | 0.95 | 1.04 |  | 10141769 | 1.33 | 1.01 | 1.74 |
| **Leucocyte formula**** |  |  |  |  |  |  |  |  |  |
| WBC (1.8 IQR 10^3^/μL) | 9423385 | 0.94 | 0.91 | 0.98 |  | 10141769 | 1.02 | 0.76 | 1.37 |
| Neutrophils (1.3 IQR 10^3^/mL) | 9423385 | 0.95 | 0.91 | 0.98 |  | 10141769 | 0.96 | 0.72 | 1.28 |
| Lymphocytes (0.64 IQR 10^3^/mL) | 9423385 | 0.99 | 0.95 | 1.03 |  | 10141769 | 1.17 | 0.88 | 1.55 |
| Monocytes (0.17 IQR 10^3^/μL) | 9423385 | 0.97 | 0.93 | 1.01 |  | 10141769 | 0.99 | 0.74 | 1.33 |
| Eosinophils (0.13 IQR 10^3^/μL) | 9423385 | 0.98 | 0.95 | 1.01 |  | 10141769 | 0.98 | 0.76 | 1.26 |
| Basophils (0.04 IQR 10^3^/mL) | 9423385 | 0.97 | 0.94 | 1.01 |  | 10141769 | 0.89 | 0.67 | 1.18 |
| **Blood parameters**** |  |  |  |  |  |  |  |  |  |
| Total Protein (0.5 IQR g/dL) | 9423385 | 1.01 | 0.97 | 1.06 |  | 10141769 | 1.05 | 0.78 | 1.43 |
| Total Colesterol (46 IQR mg/dL) | 9423385 | 1.00 | 0.96 | 1.05 |  | 10141769 | 1.22 | 0.88 | 1.68 |
| Colesterol HDL (18 IQR mg/dL) | 9423385 | 0.92 | 0.88 | 0.97 |  | 10141769 | 0.61 | 0.42 | 0.88 |
| Triglycerides (51.5 IQR mg/dL) | 9423385 | 1.02 | 1.00 | 1.05 |  | 10141769 | 1.11 | 1.00 | 1.24 |
| Ferritin (46.73 IQR ng/mL) | 9423385 | 1.03 | 1.00 | 1.06 |  | 10141769 | 1.05 | 0.86 | 1.29 |
| Glycaemia (12 IQR mg/dL) | 9423385 | 0.97 | 0.94 | 1.01 |  | 10141769 | 1.11 | 0.98 | 1.27 |
| ALT/SGPT (11 IQR U/L) | 9423385 | 1.01 | 0.98 | 1.04 |  | 10141769 | 1.05 | 0.98 | 1.13 |
| Creatinin (0.2 IQR mg/dL) | 9423385 | 1.06 | 1.00 | 1.12 |  | 10141769 | 1.24 | 0.86 | 1.77 |
| **ABO** |  |  |  |  |  |  |  |  |  |
| 0 | 4327046 | 1 |  |  |  | 4636194 | 1 |  |  |
| A | 3717298 | 1.03 | 0.96 | 1.11 |  | 4026313 | 2.03 | 1.22 | 3.39 |
| AB | 393725 | 0.97 | 0.82 | 1.15 |  | 424943 | 1.92 | 0.66 | 5.56 |
| B | 967766 | 0.97 | 0.87 | 1.09 |  | 1035079 | 0.99 | 0.38 | 2.60 |
| Missing | 17550 | 1.09 | 0.55 | 2.19 |  | 19240 | 0.00 | . | . |
| **Rh** |  |  |  |  |  |  |  |  |  |
| Neg | 1415952 | 1.02 | 0.93 | 1.12 |  | 1522883 | 0.74 | 0.37 | 1.48 |
| Pos | 7989143 | 1 |  |  |  | 8598906 | 1 |  |  |
| Missing | 18290 | 1.03 | 0.51 | 2.06 |  | 19980 | 0.00 | . | . |
| **Rh blood group antigens** |  |  |  |  |  |  |  |  |  |
| CCDEe | 19690 | 1.53 | 0.89 | 2.65 |  | 22200 | 4.20E-19 | . | . |
| CCDee | 2228201 | 1.04 | 0.95 | 1.13 |  | 2394548 | 0.97 | 0.52 | 1.81 |
| CCDuee | 1480 | 1.09E-19 | . | . |  | 1480 | 6.97E-18 | . | . |
| CcDEE | 6660 | 2.78E-20 | . | . |  | 6660 | 2.05E-18 | . | . |
| CcDEe | 1121962 | 1.02 | 0.92 | 1.14 |  | 1212137 | 1.19 | 0.58 | 2.48 |
| CcDee | 3347239 | 1 |  |  |  | 3592975 | 1 |  |  |
| CcDuee | 41688 | 0.80 | 0.46 | 1.38 |  | 44400 | 1.84E-19 | . | . |
| CcdEe | 1417 | 1.82 | 0.26 | 12.96 |  | 1480 | 1.68E-17 | . | . |
| Ccdee | 57450 | 1.15 | 0.78 | 1.71 |  | 63640 | 1.65E-19 | . | . |
| ccDEE | 128486 | 1.34 | 1.04 | 1.72 |  | 143501 | 0.94 | 0.13 | 6.95 |
| ccDEe | 882029 | 1.03 | 0.92 | 1.16 |  | 949832 | 1.47 | 0.71 | 3.05 |
| ccDee | 204660 | 1.02 | 0.82 | 1.28 |  | 223773 | 0.64 | 0.09 | 4.75 |
| ccDuEe | 2220 | 6.23E-20 | . | . |  | 2220 | 1.06E-17 | . | . |
| ccDuee | 4170 | 0.55 | 0.08 | 3.90 |  | 4440 | 3.05E-18 | . | . |
| ccdEe | 24549 | 1.11 | 0.60 | 2.07 |  | 25823 | 4.76 | 0.64 | 35.19 |
| ccdee | 1331796 | 1.04 | 0.94 | 1.15 |  | 1431200 | 0.73 | 0.33 | 1.61 |
| Missing | 19688 | 1.11 | 0.58 | 2.14 |  | 21460 | 0.00 | . | . |
| **Kell** |  |  |  |  |  |  |  |  |  |
| K+ | 20068 | 0.77 | 0.35 | 1.71 |  | 21460 | 5.54E-19 | . | . |
| KK | 19055 | 1.25 | 0.67 | 2.33 |  | 19980 | 4.50E-19 | . | . |
| Kk | 772065 | 0.95 | 0.84 | 1.07 |  | 824206 | 0.81 | 0.33 | 2.01 |
| kk | 8593249 | 1 |  |  |  | 9255403 | 1 |  |  |
| Missing | 18948 | 1.14 | 0.59 | 2.20 |  | 20720 | 0.00 | . | . |
| **Cw*** |  |  |  |  |  |  |  |  |  |
| Cw+ | 71320 | 1.04 | 0.71 | 1.51 |  | 76960 | 9.14E-20 | . | . |
| Cw- | 3288204 | 1 |  |  |  | 3530512 | 1 |  |  |
| **MN*** |  |  |  |  |  |  |  |  |  |
| MN | 2324929 | 1 |  |  |  | 2499225 | 1 |  |  |
| NN | 953655 | 0.95 | 0.84 | 1.07 |  | 1022272 | 0.75 | 0.28 | 2.06 |
| MM | 1563792 | 0.95 | 0.86 | 1.05 |  | 1672713 | 1.04 | 0.48 | 2.24 |
| **Ss*** |  |  |  |  |  |  |  |  |  |
| SS | 667318 | 1.15 | 1.00 | 1.32 |  | 717574 | 1.17 | 0.42 | 3.22 |
| Ss | 2230864 | 1 |  |  |  | 2376994 | 1 |  |  |
| ss | 1900289 | 1.15 | 1.04 | 1.27 |  | 2050866 | 0.86 | 0.39 | 1.87 |
| **Duffy*** |  |  |  |  |  |  |  |  |  |
| Fya-b+ | 1781473 | 1 |  |  |  | 1901915 | 1 |  |  |
| Fya+b+ | 2124343 | 1.03 | 0.93 | 1.15 |  | 2280069 | 0.49 | 0.23 | 1.08 |
| Fya+b- | 882930 | 1.07 | 0.94 | 1.22 |  | 953026 | 0.60 | 0.22 | 1.63 |
| Fya-b- | 18557 | 0.74 | 0.33 | 1.66 |  | 19980 | 7.52E-19 | . | . |
| **Kidd*** |  |  |  |  |  |  |  |  |  |
| Jka-b+ | 1171802 | 0.95 | 0.85 | 1.07 |  | 1253464 | 2.12 | 0.95 | 4.72 |
| Jka+b+ | 2401781 | 1 |  |  |  | 2578289 | 1 |  |  |
| Jka+b- | 1226353 | 0.99 | 0.88 | 1.10 |  | 1313617 | 1.27 | 0.52 | 3.10 |
| Jka-b- | 1480 | 8.04E-20 | . | . |  | 1480 | 2.27E-17 | . | . |

NOTES:

* Tested only on O-group donors

** HRs for interquartile range (IQR) increases (equal to the difference between the 25th and 75th percentile)

*Persons-days* = the sum of the time each person was positive, added for all persons
