# Supplementary material for: A cohort study on the biochemical and haematological parameters of Italian blood donors as possible risk factors of COVID-19 infection and severe disease in the pre- and post-Omicron period
Source: PLoS One. 2023 Nov 21;18(11):e0294272. doi: 10.1371/journal.pone.0294272 (PMC10662768; doi:10.1371/journal.pone.0294272)
Supplement: S2 Table — (DOCX) [file pone.0294272.s002.docx]

**S2 Table. Cox proportional regression analysis adjusted by sex, age, and vaccination status in donors tested positive for SARS-CoV-2 before and after December 20, 2021 (pre- and post-Omicron diffusion, respectively):**

|  | **Positive for SARS-CoV-2** | | | | | | | | | | |
| --- | --- | --- | --- | --- | --- | --- | --- | --- | --- | --- | --- |
|  | **Period of infection from Feb 20, 2020 to Dec 20, 2021** | | | | |  | **Period of infection from Jan 01, 2022 to Feb 28, 2022** | | | | |
|  | **Persons-days** | **Infections** | **HR** | **95%CI** | |  | **Persons-days** | **Infections** | **HR** | **95%CI** | |
| **Sex** |  |  |  |  |  |  |  |  |  |  |  |
| **Males** | 5993665 | 1189 | 1 |  |  |  | 439549 | 1169 | 1 |  |  |
| **Females** | 2662085 | 488 | 0.93 | 0.84 | 1.04 |  | 196381 | 510 | 0.97 | 0.87 | 1.08 |
| **Age** | 8655750 | 1677 | 0.99 | 0.99 | 1.00 |  | 635930 | 1679 | 0.99 | 0.99 | 0.99 |
| **Vaccination status** |  |  |  |  |  |  |  |  |  |  |  |
| Unvaccinated | 6298795 | 1458 | 1 |  |  |  | 34378 | 213 | 1 |  |  |
| Vaccinated with one dose | 525526 | 45 | 0.57 | 0.41 | 0.79 |  | 4888 | 27 | 0.81 | 0.54 | 1.21 |
| Vaccinated with two doses | 1774292 | 171 | 0.26 | 0.20 | 0.34 |  | 157220 | 805 | 0.70 | 0.60 | 0.81 |
| Vaccinated with three doses | 57137 | 3 | 0.03 | 0.01 | 0.11 |  | 439444 | 634 | 0.28 | 0.24 | 0.33 |
| **Blood count**** |  |  |  |  |  |  |  |  |  |  |  |
| RBC (0.6 IQR 10^6^//μl) | 8655750 | 1677 | 1.05 | 0.97 | 1.13 |  | 635930 | 1679 | 0.97 | 0.89 | 1.05 |
| HGB (1.7 IQR g/dL) | 8655750 | 1677 | 0.99 | 0.91 | 1.08 |  | 635930 | 1679 | 0.96 | 0.88 | 1.05 |
| MCV (5 IQR fL) | 8655750 | 1677 | 0.93 | 0.87 | 0.98 |  | 635930 | 1679 | 0.97 | 0.91 | 1.03 |
| MCH (1.9 IQR pg) | 8655750 | 1677 | 0.97 | 0.95 | 1.00 |  | 635930 | 1679 | 1.00 | 0.95 | 1.06 |
| MCHC (1.3 IQR g/dL) | 8655750 | 1677 | 1.01 | 0.95 | 1.07 |  | 635930 | 1679 | 1.05 | 0.99 | 1.11 |
| RDW (0.9 IQR %) | 8655750 | 1677 | 0.96 | 0.90 | 1.01 |  | 635930 | 1679 | 0.97 | 0.92 | 1.02 |
| PLT (59 IQR 10^3^/μL) | 8655750 | 1677 | 0.98 | 0.92 | 1.04 |  | 635930 | 1679 | 0.99 | 0.93 | 1.06 |
| MPV (1.6 IQR fL) | 8655750 | 1677 | 0.99 | 0.93 | 1.06 |  | 635930 | 1679 | 1.01 | 0.95 | 1.08 |
| **Leucocyte formula**** |  |  |  |  |  |  |  |  |  |  |  |
| WBC (1.8 IQR 10^3^/μL) | 8655750 | 1677 | 0.95 | 0.89 | 1.01 |  | 635930 | 1679 | 0.96 | 0.90 | 1.02 |
| Neutrophils (1.3 IQR 10^3^/mL) | 8655750 | 1677 | 0.96 | 0.91 | 1.02 |  | 635930 | 1679 | 0.95 | 0.90 | 1.01 |
| Lymphocytes (0.64 IQR 10^3^/mL) | 8655750 | 1677 | 0.97 | 0.91 | 1.03 |  | 635930 | 1679 | 0.99 | 0.93 | 1.05 |
| Monocytes (0.17 IQR 10^3^/μL) | 8655750 | 1677 | 0.94 | 0.89 | 1.01 |  | 635930 | 1679 | 1.00 | 0.94 | 1.06 |
| Eosinophils (0.13 IQR 10^3^/μL) | 8655750 | 1677 | 0.95 | 0.91 | 1.01 |  | 635930 | 1679 | 1.01 | 0.96 | 1.06 |
| Basophils (0.04 IQR 10^3^/mL) | 8655750 | 1677 | 0.95 | 0.89 | 1.00 |  | 635930 | 1679 | 0.99 | 0.94 | 1.04 |
| **Blood parameters**** |  |  |  |  |  |  |  |  |  |  |  |
| Creatinin (0.2 IQR mg/dL) | 8655750 | 1677 | 1.06 | 0.98 | 1.14 |  | 635930 | 1679 | 1.05 | 0.97 | 1.13 |
| **ABO** |  |  |  |  |  |  |  |  |  |  |  |
| 0 | 3960224 | 694 | 1 |  |  |  | 290360 | 820 | 1 |  |  |
| A | 3401375 | 727 | 1.22 | 1.10 | 1.36 |  | 250222 | 617 | 0.89 | 0.80 | 0.98 |
| AB | 357851 | 76 | 1.22 | 0.96 | 1.54 |  | 26767 | 64 | 0.86 | 0.67 | 1.12 |
| B | 884632 | 163 | 1.04 | 0.88 | 1.24 |  | 64850 | 173 | 0.92 | 0.78 | 1.09 |
| Missing | 51668 | 17 | 1.93 | 1.19 | 3.12 |  | 3731 | 5 | 0.51 | 0.21 | 1.24 |
| **Cw*** |  |  |  |  |  |  |  |  |  |  |  |
| Cw+ | 65553 | 16 | 1.32 | 0.80 | 2.17 |  | 4799 | 11 | 0.88 | 0.48 | 1.59 |
| Cw- | 3019809 | 553 | 1 |  |  |  | 222219 | 599 | 1 |  |  |
| **MN*** |  |  |  |  |  |  |  |  |  |  |  |
| MN | 2135274 | 393 | 1 |  |  |  | 156993 | 427 | 1 |  |  |
| NN | 874992 | 153 | 0.95 | 0.79 | 1.15 |  | 65210 | 168 | 0.94 | 0.79 | 1.12 |
| MM | 1435308 | 257 | 0.97 | 0.83 | 1.13 |  | 106526 | 269 | 0.92 | 0.79 | 1.07 |

NOTES:

* Tested only on O-group donors

** HRs for interquartile range (IQR) increases (equal to the difference between the 25th and 75th percentile)

*Persons-days* = the sum of the time each person was positive, added for all persons
