# Supplementary material for: A cohort study on the biochemical and haematological parameters of Italian blood donors as possible risk factors of COVID-19 infection and severe disease in the pre- and post-Omicron period
Source: PLoS One. 2023 Nov 21;18(11):e0294272. doi: 10.1371/journal.pone.0294272 (PMC10662768; doi:10.1371/journal.pone.0294272)
Supplement: S3 Table — (DOCX) [file pone.0294272.s003.docx]

**S3 Table. Cox proportional regression analysis adjusted by age, sex, and vaccination status in male and female donors:**

|  |  | **Positive for SARS-CoV-2 - Period of infection from Feb 20, 2020 to Feb 28, 2022** | | | | | | | | | | | |
| --- | --- | --- | --- | --- | --- | --- | --- | --- | --- | --- | --- | --- | --- |
|  |  | **Male** | | | | | |  | **Female** | | | | |
|  | **Persons-days** | | **Infections** | **HR** |  | **95%CI** | |  | **Persons-days** | **Infections** | **HR** | **95%CI** | |
| **Age** | 6524180 | | 2574 | 0.99 |  | 0.99 | 1.00 |  | 2899205 | 1116 | 0.99 | 0.98 | 0.99 |
| **Vaccination status** |  | |  |  |  |  |  |  |  |  |  |  |  |
| Unvaccinated | 4428583 | | 1207 | 1 |  |  |  |  | 1913318 | 502 | 1 |  |  |
| Vaccinated with one dose | 363995 | | 59 | 0.85 |  | 0.64 | 1.12 |  | 168348 | 20 | 0.52 | 0.33 | 0.83 |
| Vaccinated with two doses | 1372461 | | 854 | 0.58 |  | 0.50 | 0.66 |  | 642543 | 360 | 0.54 | 0.44 | 0.67 |
| Vaccinated with three doses | 359141 | | 454 | 0.23 |  | 0.19 | 0.26 |  | 174996 | 234 | 0.25 | 0.19 | 0.31 |
| **Blood count**** |  | |  |  |  |  |  |  |  |  |  |  |  |
| WBC (1.8 IQR 10^3^/μL) | 6524180 | | 2574 | 0.92 |  | 0.88 | 0.97 |  | 2899205 | 1116 | 0.98 | 0.91 | 1.05 |
| RBC (0.6 IQR 10^6^//μl)) | 6524180 | | 2574 | 0.98 |  | 0.92 | 1.04 |  | 2899205 | 1116 | 1.03 | 0.93 | 1.15 |
| HGB (1.7 IQR g/dL) | 6524180 | | 2574 | 0.97 |  | 0.90 | 1.04 |  | 2899205 | 1116 | 0.96 | 0.86 | 1.08 |
| MCV (5 IQR fL) | 6524180 | | 2574 | 0.97 |  | 0.93 | 1.02 |  | 2899205 | 1116 | 0.94 | 0.88 | 1.01 |
| MCH (1.9 IQR pg) | 6524180 | | 2574 | 1.00 |  | 0.96 | 1.04 |  | 2899205 | 1116 | 0.95 | 0.90 | 1.01 |
| MCHC (1.3 IQR g/dL) | 6524180 | | 2574 | 1.04 |  | 0.99 | 1.09 |  | 2899205 | 1116 | 1.00 | 0.93 | 1.07 |
| RDW (0.9 IQR %) | 6524180 | | 2574 | 0.95 |  | 0.90 | 0.99 |  | 2899205 | 1116 | 0.97 | 0.91 | 1.03 |
| PLT (59 IQR 103/μL) | 6524180 | | 2574 | 0.93 |  | 0.88 | 0.98 |  | 2899205 | 1116 | 1.06 | 0.99 | 1.14 |
| MPV (1.6 IQR fL) | 6524180 | | 2574 | 1.03 |  | 0.98 | 1.09 |  | 2899205 | 1116 | 0.92 | 0.85 | 0.99 |
| **Leucocyte formula**** |  | |  |  |  |  |  |  |  |  |  |  |  |
| Neutrophils (1.3 IQR 103/mL) | 6524180 | | 2574 | 0.94 |  | 0.89 | 0.98 |  | 2899205 | 1116 | 0.96 | 0.90 | 1.02 |
| Lymphocytes (0.64 IQR 103/mL) | 6524180 | | 2574 | 0.96 |  | 0.91 | 1.01 |  | 2899205 | 1116 | 1.04 | 0.97 | 1.12 |
| Monocytes (0.17 IQR 10^3^/μL) | 6524180 | | 2574 | 0.95 |  | 0.91 | 1.00 |  | 2899205 | 1116 | 1.00 | 0.93 | 1.08 |
| Eosinophils (0.13 IQR 10^3^/μL) | 6524180 | | 2574 | 0.96 |  | 0.92 | 1.00 |  | 2899205 | 1116 | 1.03 | 0.97 | 1.09 |
| Basophils (0.04 IQR 103/mL) | 6524180 | | 2574 | 0.97 |  | 0.93 | 1.01 |  | 2899205 | 1116 | 0.97 | 0.91 | 1.04 |
| **Blood parameters**** |  | |  |  |  |  |  |  |  |  |  |  |  |
| Creatinin (0.2 IQR mg/dL) | 6524180 | | 2574 | 1.08 |  | 1.01 | 1.15 |  | 2899205 | 1116 | 1.00 | 0.90 | 1.12 |
| **ABO** |  | |  |  |  |  |  |  |  |  |  |  |  |
| 0 | 2957711 | | 1188 | 1 |  |  |  |  | 1353504 | 491 | 1 |  |  |
| A | 2591951 | | 1017 | 0.98 |  | 0.90 | 1.07 |  | 1111112 | 457 | 1.15 | 1.01 | 1.30 |
| AB | 281957 | | 105 | 0.92 |  | 0.75 | 1.12 |  | 108068 | 42 | 1.15 | 0.84 | 1.57 |
| B | 662610 | | 246 | 0.91 |  | 0.79 | 1.04 |  | 300338 | 121 | 1.10 | 0.90 | 1.34 |
| Missing | 29951 | | 18 | 1.63 |  | 1.02 | 2.59 |  | 26183 | 5 | 0.56 | 0.23 | 1.34 |
| **Cw*** |  | |  |  |  |  |  |  |  |  |  |  |  |
| Cw+ | 53476 | | 19 | 0.92 |  | 0.58 | 1.45 |  | 17844 | 9 | 1.44 | 0.74 | 2.79 |
| Cw- | 2371799 | | 929 | 1 |  |  |  |  | 916405 | 336 | 1 |  |  |
| **MN*** |  | |  |  |  |  |  |  |  |  |  |  |  |
| MN | 1744314 | | 680 | 1 |  |  |  |  | 580615 | 219 | 1 |  |  |
| NN | 730042 | | 285 | 1.00 |  | 0.87 | 1.14 |  | 223613 | 66 | 0.77 | 0.59 | 1.01 |
| MM | 1187681 | | 448 | 0.97 |  | 0.86 | 1.09 |  | 376111 | 131 | 0.90 | 0.72 | 1.11 |

NOTES:

* Tested only on O-group donors

** HRs for interquartile range (IQR) increases (equal to the difference between the 25th and 75th percentile)

*Persons-days* = the sum of the time each person was positive, added for all persons
