# Supplementary material for: A cohort study on the biochemical and haematological parameters of Italian blood donors as possible risk factors of COVID-19 infection and severe disease in the pre- and post-Omicron period
Source: PLoS One. 2023 Nov 21;18(11):e0294272. doi: 10.1371/journal.pone.0294272 (PMC10662768; doi:10.1371/journal.pone.0294272)
Supplement: S4 Table — (DOCX) [file pone.0294272.s004.docx]

**S4 Table. Cox proportional regression analysis adjusted by age, sex, and vaccination status in donors under (<50) and over (>=50) the age of 50:**

|  | **Positive for SARS-CoV-2 - Period of infection from Feb 20, 2020 to Feb 28, 2022** | | | | | | | | | | |
| --- | --- | --- | --- | --- | --- | --- | --- | --- | --- | --- | --- |
|  | **<50** | | | | |  | **>=50** | | | | |
|  | **Persons-days** | **Infections** | **HR** | **95%CI** | |  | **Persons-days** | **Infections** | **HR** | **95%CI** | |
| **Males** | 3925319 | 1739 | 1 |  |  |  | 2598861 | 835 | 1 |  |  |
| **Females** | 1862346 | 818 | 1.02 | 0.94 | 1.11 |  | 1036859 | 298 | 0.91 | 0.80 | 1.04 |
| **Vaccination status** |  |  |  |  |  |  |  |  |  |  |  |
| Unvaccinated | 3990391 | 1124 | 1 |  |  |  | 2351510 | 585 | 1 |  |  |
| Vaccinated with one dose | 303461 | 55 | 0.78 | 0.58 | 1.03 |  | 228882 | 24 | 0.63 | 0.41 | 0.97 |
| Vaccinated with two doses | 1200389 | 897 | 0.62 | 0.54 | 0.71 |  | 814615 | 317 | 0.46 | 0.37 | 0.58 |
| Vaccinated with three doses | 293424 | 481 | 0.28 | 0.24 | 0.33 |  | 240713 | 207 | 0.16 | 0.12 | 0.20 |
| **Blood count**** |  |  |  |  |  |  |  |  |  |  |  |
| WBC (1.8 IQR 10^3^/μL) | 5787665 | 2557 | 0.97 | 0.92 | 1.02 |  | 3635720 | 1133 | 0.88 | 0.81 | 0.95 |
| RBC (0.6 IQR 10^6^//μl)) | 5787665 | 2557 | 0.99 | 0.93 | 1.06 |  | 3635720 | 1133 | 1.00 | 0.91 | 1.10 |
| HGB (1.7 IQR g/dL) | 5787665 | 2557 | 0.95 | 0.88 | 1.02 |  | 3635720 | 1133 | 1.00 | 0.90 | 1.11 |
| MCV (5 IQR fL) | 5787665 | 2557 | 0.96 | 0.92 | 1.01 |  | 3635720 | 1133 | 0.96 | 0.89 | 1.03 |
| MCH (1.9 IQR pg) | 5787665 | 2557 | 0.97 | 0.93 | 1.01 |  | 3635720 | 1133 | 1.00 | 0.94 | 1.07 |
| MCHC (1.3 IQR g/dL) | 5787665 | 2557 | 1.01 | 0.96 | 1.05 |  | 3635720 | 1133 | 1.07 | 0.99 | 1.15 |
| RDW (0.9 IQR %) | 5787665 | 2557 | 0.97 | 0.93 | 1.01 |  | 3635720 | 1133 | 0.93 | 0.87 | 0.99 |
| PLT (59 IQR 103/μL) | 5787665 | 2557 | 1.00 | 0.95 | 1.05 |  | 3635720 | 1133 | 0.92 | 0.85 | 0.99 |
| MPV (1.6 IQR fL) | 5787665 | 2557 | 0.99 | 0.94 | 1.04 |  | 3635720 | 1133 | 1.02 | 0.94 | 1.10 |
| **Leucocyte formula**** |  |  |  |  |  |  |  |  |  |  |  |
| Neutrophils (1.3 IQR 103/mL) | 5787665 | 2557 | 0.97 | 0.93 | 1.01 |  | 3635720 | 1133 | 0.87 | 0.81 | 0.94 |
| Lymphocytes (0.64 IQR 103/mL) | 5787665 | 2557 | 1.00 | 0.95 | 1.05 |  | 3635720 | 1133 | 0.97 | 0.90 | 1.05 |
| Monocytes (0.17 IQR 10^3^/μL) | 5787665 | 2557 | 0.98 | 0.94 | 1.03 |  | 3635720 | 1133 | 0.94 | 0.87 | 1.01 |
| Eosinophils (0.13 IQR 10^3^/μL) | 5787665 | 2557 | 0.98 | 0.94 | 1.02 |  | 3635720 | 1133 | 0.99 | 0.93 | 1.05 |
| Basophils (0.04 IQR 103/mL) | 5787665 | 2557 | 0.97 | 0.93 | 1.01 |  | 3635720 | 1133 | 0.97 | 0.91 | 1.04 |
| **Blood parameters**** |  |  |  |  |  |  |  |  |  |  |  |
| Creatinin (0.2 IQR mg/dL) | 5787665 | 2557 | 1.00 | 0.93 | 1.06 |  | 3635720 | 1133 | 1.19 | 1.09 | 1.31 |
| **ABO** |  |  |  |  |  |  |  |  |  |  |  |
| 0 | 2664892 | 1188 | 1 |  |  |  | 1646323 | 491 | 1 |  |  |
| A | 2266269 | 1012 | 1.01 | 0.93 | 1.10 |  | 1436794 | 462 | 1.07 | 0.94 | 1.22 |
| AB | 229825 | 99 | 0.97 | 0.79 | 1.19 |  | 160200 | 48 | 1.02 | 0.76 | 1.37 |
| B | 597335 | 246 | 0.92 | 0.80 | 1.06 |  | 365613 | 121 | 1.08 | 0.89 | 1.32 |
| Missing | 29344 | 12 | 0.94 | 0.53 | 1.67 |  | 26790 | 11 | 1.47 | 0.81 | 2.67 |
| **Cw*** |  |  |  |  |  |  |  |  |  |  |  |
| Cw+ | 49783 | 23 | 1.17 | 0.77 | 1.77 |  | 21537 | 5 | 0.67 | 0.28 | 1.61 |
| Cw- | 2169611 | 920 | 1 |  |  |  | 1118593 | 345 | 1 |  |  |
| **MN** |  |  |  |  |  |  |  |  |  |  |  |
| MN | 1356522 | 607 | 1 |  |  |  | 968407 | 292 | 1 |  |  |
| NN | 557025 | 233 | 0.93 | 0.80 | 1.08 |  | 396630 | 118 | 0.97 | 0.79 | 1.21 |
| MM | 934155 | 385 | 0.91 | 0.80 | 1.03 |  | 629637 | 194 | 1.04 | 0.87 | 1.25 |

NOTES:

* Tested only on O-group donors

** HRs for interquartile range (IQR) increases (equal to the difference between the 25th and 75th percentile)

*Persons-days* = the sum of the time each person was positive, added for all persons
